# Renoprotective effect of local sildenafil administration in renal ischaemia–reperfusion injury: A randomised controlled canine study

**DOI:** 10.1080/2090598X.2019.1600995

**Published:** 2019-04-18

**Authors:** Mohamed H. Zahran, Nashwa Barakat, Shery Khater, Amira Awadalla, Ahmed Mosbah, Adel Nabeeh, Abdelaziz M. Hussein, Ahmed A. Shokeir

**Affiliations:** a Urology and Nephrology Center, Mansoura University, Mansoura, Egypt; b Medical Physiology Department, Faculty of Medicine, Mansoura University, ‎Mansoura, Egypt

**Keywords:** Acute kidney injury, ischaemia reperfusion injury, oxidative stress, apoptosis, sildenafil

## Abstract

**Objectives**: To design a new canine model to assess the renoprotective effect of local sildenafil administration, as the renoprotective effect of systemic sildenafil administration in renal ischaemia–reperfusion (IR) injury in animal models has been shown but its local effects have not been established to date.

**Materials and methods**: In all, 120 dogs were assigned to five groups: sham, oral control (OC) group (right nephrectomy + left renal ischaemia for 60 min), oral sildenafil (OS) group (oral sildenafil 1 mg/kg, 60 min before ischaemia), local control (LC) group (local renal perfusion with saline and heparin for 5 min) and local sildenafil (LS) group (perfusion with sildenafil 0.5 mg/kg). Renal functions, histopathological changes, expression of caspase-3, nuclear factor erythroid 2-related factor 2 (Nrf2), inflammatory cytokines (intracellular adhesion molecule 1, tumour necrosis factor α and interleukin 1β) and endothelial nitric oxide synthase (eNOS) in renal tissues were assessed in all groups at 1, 3, 7 and 14 days.

**Results**: There were significant improvements in renal functions and cortical and medullary damage scores in the sildenafil-treated groups compared to their control groups (*P* < 0.05). Also, the LS group showed significantly better improvement of renal functions and cortical and medullary damage scores than the OS group (*P* < 0.05). Moreover, sildenafil significantly decreased the expression of caspase-3 and inflammatory cytokines and increased the expression of Nrf2 and eNOS in renal tissue, which were statistically significant in the LS group.

**Conclusion**: LS has a greater renoprotective effect against renal IR injury than systemic administration via anti-inflammatory, antioxidant and anti-apoptotic pathways.

**Abbreviations**: BUN: blood urea nitrogen; Ct: cycle threshold; eNOS: endothelial nitric oxide synthase; GAPDH: glyceraldehyde 3-phosphate dehydrogenase; H&E: haematoxylin and eosin; IL-1β: interleukin 1β; NO: nitric oxide; Nrf2: nuclear factor erythroid 2-related factor 2; OC: oral control; OS: oral sildenafil; LC: local control; LS: local sildenafil

## Introduction

Since the first successful kidney transplantation in 1954, there has been a rapid growth in publications dealing with care of the donated kidney. Although special care in donor management, use of cold ischaemia, the introduction of better preservation solutions in clinical practice, and shortening cold ischaemic time have been implemented, ischaemia–reperfusion (IR) injury remains a major problem in renal transplantation [1]. Renal IR injury is a complex pathological condition associated with significant clinical consequences. It is a common cause of acute kidney injury, delayed graft function, as well as development of interstitial fibrosis that affects intermediate- and long-term graft function and survival [2]. The continuous improvement in experimental models has allowed better understanding of the multifaceted and interacting mechanisms of IR injury, focussing on certain mechanisms involved in the regulation of renal function and identifying new effective drugs that modify the course and improve recovery from renal IR injury. Many pharmacological and non-pharmacological interventions have been investigated for their ability to reduce renal IR injury in animal models in order to be applied clinically [3,4]. However, a definitive treatment of renal IR injury has not been determined to date.

Sildenafil is a well-known drug used for treatment of pulmonary hypertension and male erectile dysfunction due to its vasodilatory effect. It mediates its effect via inhibition of phosphodiesterase 5 enzyme with subsequent up-regulation of cGMP and nitric oxide (NO) [5]. A few studies have examined the role of systemic sildenafil in renal protection against IR injury. Some reported improved renal haemodynamics after relief of ischaemia [6,7]. Others confirmed its renoprotective effect in renal IR injury in rats [5,8–10]. It exerts its action via anti-inflammatory, anti-apoptotic, and antioxidant mechanisms [5]. Nevertheless, the systemic use of sildenafil is associated with hypotension that adversely affects renal perfusion after relief of ischaemia [11]. Also, the local effect of sildenafil on IR injury has not been established to date.

In the present study, we designed a new canine model to assess the renoprotective effect of local sildenafil (LS) administration in renal perfusion in a large animal (dog) in a manner mimicking the process of renal perfusion in live-donor renal transplantation and compared it with the already proven systemic effect.

## Materials and methods

This is a randomised controlled experimental study including 120 mongrel dogs aged 2–3 years, weighing 18–25 kg. Dogs were housed in the animal research facility in the Urology and Nephrology Center at Mansoura, Egypt. The experiment was carried out according to the guidelines for the Care and Use of Laboratory Animals and the research was approved by the Local Ethics Committee of the Faculty of Medicine, Mansoura University, Egypt (Mansoura Faculty of Medicine- Institutional Research Board MFM-IRB) and given the number (# MD/41.r).

### IR injury model

**Figure 1.**
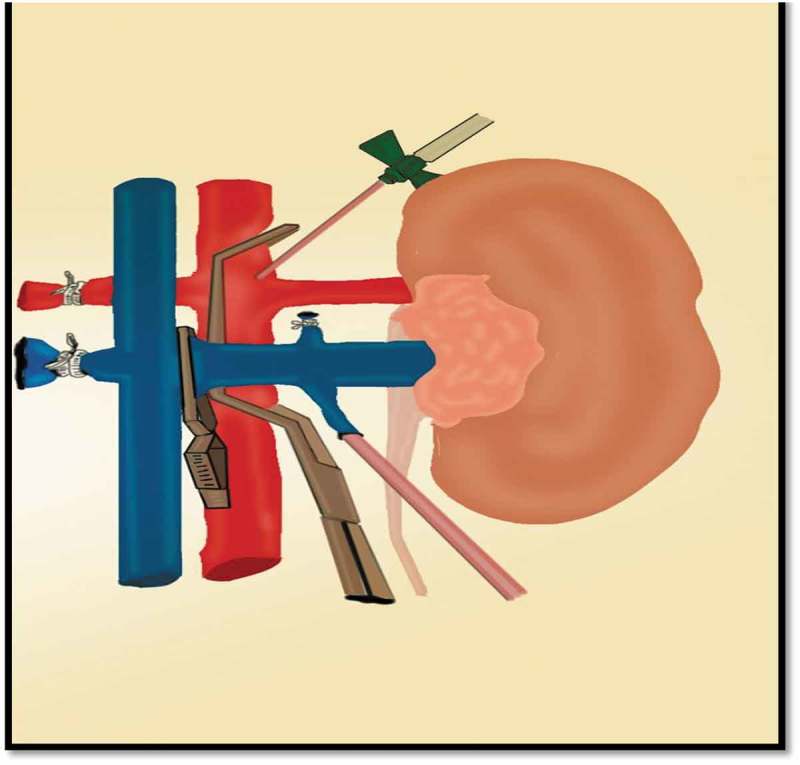
Model of renal ischaemia with local perfusion. The Stansky clamp partially occludes the aortic lumen at the level of renal artery. A cannula is inserted in the arteriotomy to deliver the perfusion solution. After clamping of the renal vein with a small Bulldog clamp, a cannula is inserted through the proximal end of the gonadal vein to carry out the perfusion solution outside the abdomen.

Dogs were anaesthetised with thiopental sodium (10 mg/kg) for induction, followed by endotracheal intubation. Anaesthesia was maintained with oxygen and air by a ventilator. The saphenous vein in the hind-limb was cannulated for drugs and fluid infusion. All the dogs received i.v. chemoprophylaxis (cefotaxime 50 mg/kg) preoperatively. The skin of the abdomen was disinfected with Betadine solution and the procedure was performed under sterile conditions. A warm ischaemia model was applied using a midline laparotomy incision. The left kidney was dissected off of its surrounding perirenal fat along the renal surface, and then the left renal artery and vein were dissected. Ischaemia of the left kidney for 60 min was achieved by applying a Satinsky vascular clamp on the aorta at the site of the renal artery origin. Then, the left renal vein was clamped distal to the origin of the gonadal vein. The gonadal vein was dissected, the distal end was ligated and the proximal end was ligated over a vascular cannula. After clamping, an arteriotomy was done in the aorta. Perfusion of the ischaemic kidney with saline 0.9% mixed with heparin through the arteriotomy was done under gravity and the perfusion was drained through the cannula inserted through the gonadal vein to outside the abdominal cavity. The process of perfusion was maintained until the kidney became blanched and the effluent became clear. The arteriotomy was sutured using 5/0 polypropylene (Prolene®; Ethicon Inc., Somerville, NJ, USA) sutures and the cannula was removed, and the proximal end of the gonadal vein was ligated using 3/0 polyglactin 910 (Vicryl®; Ethicon Inc.) suture. At 5 min before release of the Satinsky vascular clamp, the vascular pedicle of the right kidney was exposed and ligated using 3–0 silk sutures twice and the right kidney was removed. The abdomen was irrigated with isotonic saline and the abdominal incision was closed by continuous sutures using 2/0 polyglactin 910 suture (Figure 1). After surgery, the dogs were allowed free access to food and water.

### Study design

The dogs were randomly allocated into five groups (24 dogs in each): a sham group, the left renal pedicle was exposed without ischaemia with right nephrectomy; oral control (OC) group, right nephrectomy and left renal ischaemia for 60 min; oral sildenafil (OS) group, as OC plus pretreatment with oral sildenafil (Viagra; Pfizer, Egypt) 1 mg/kg body weight 1 h before ischaemia; local control (LC) group, as OC group plus perfusion of the left kidney with saline 0.9% and unfractionated heparin 5000 IU; and LS group: as LC group with addition of sildenafil (Revatio; Pfizer, Sandwich, UK) 0.5 mg/kg body weight in the perfusion fluid. Each group was further subdivided into four subgroups (six dogs in each) according to the time of death, i.e. days 1, 3, 7 and 14.

### Collections of blood samples and harvesting of kidney specimens

Blood samples (3 mL) were taken from the saphenous vein in the hind limb before the procedure (basal) and at the end of experiment. The collected blood was centrifuged and serum was obtained and stored at – 80ºC for measurement of serum creatinine and blood urea nitrogen (BUN). Also, at the end of the experiment the dogs were anaesthetised and the left kidneys were harvested and divided into two halves, one half was placed in formalin (10%) for histopathological and immunohistochemical examinations and the second half stored at – 80ºC for molecular studies.

### Assessment of renal functions

Serum creatinine and BUN were estimated from blood samples using an auto-analyser (CX7; Beckman, Foster City, CA, USA). Also, the GFR of the left kidney was assessed by mercapto-acetyltriglycine (MAG3) diuretic renography before the procedure (basal) and on the final day of study. Diuretic renography was performed according to a standard protocol [12].

### Histopathological examination

The formalin-fixed part of the left kidney was processed and paraffin blocked. Sections of 3 µm were taken and stained with haematoxylin and eosin (H&E) for assessment of glomerular and tubular injury (necrosis, casts, epithelial simplification, loss of brush borders, and cell blebs). Histopathological examination was performed by pathologists blinded to the experimental study. Acute tubular injury was assessed and scored from 0 to 3. Damage affecting ≤5% of the field was scored 0; mild damage affecting 5–25% of the field was scored 1; moderate damage affecting 25–75% of the field was scored 2; and severe damage of >75% of the field was scored 3 [13]. Masson’s trichrome staining was used to assess interstitial fibrosis. Paraffin sections were deparaffinised, stained with Weigert’s haematoxylin then stained with a solution containing chromotropic acid, light green, phosphotungstic acid, and glacial acetic acid, followed by 0.5% light green. Interstitial fibrosis was assessed in 20 fields at ×400. Interstitial fibrosis was also scored as 0 = no fibrosis, 1 = mild fibrosis, 2 = moderate fibrosis, and 3 = marked fibrosis [13].

### Immunohistochemical assay of caspase-3

Coated slides were prepared from formalin-fixed paraffin sections for immunohistochemical examination of caspase-3 expression and measuring the apoptotic index using polyclonal anti-cleaved caspase-3 antibody (1:1000; cat. no. #9661, Cell Signaling Technology, Boston, MA, USA). The apoptotic index of caspase-3 was assessed with a standard point-counting method for the percentage of labelled tubular cells (excluding necrotic tubules) in non-overlapping, randomly selected 10 high-power fields of each slide. Labelling indices were expressed as the mean scores of the 10 fields [14].

### Immunofluorescent assay of nuclear factor erythroid 2-related factor 2 (Nrf2)

Paraffin sections were rehydrated and immunofluorescent stained using Nrf-2 antibody (1:200; Dako Cytomation, Glostrup, Denmark). The scoring pattern for Nrf2 staining was as follows: score 0, negative staining for all cells; score 1+, weakly positive for staining in <10% of cells; score 2+, moderate to strong positive staining covering between 10% to 50% of cells, and score 3+, strongly positive staining including >50% cells [15].

### Assessment of markers of inflammatory cytokines and endothelial nitric oxide synthase (eNOS) by real-time PCR

Quantitative real-time PCR for the expression of mRNA of eNOS and markers of inflammation, e.g. TNF-α, interleukin 1β (IL-1β), and intracellular adhesion molecule 1 (ICAM-1), was done according to a previously described technique [5]. Primers of the tested genes included, TNF-α [5′-TACTGAACTTC GGGGTGATTGGTCC-3′ (sense), 5′-CAGCCTTGTC CCTTGAAGAGAACC-3′ (antisense)], IL-1β **[**5′-TGTGATGTTCCCATTAGAC-3′ (sense), 5′-AATA CCACTTGTTGGCTTA-3′ (antisense)], ICAM-1 [5′- AGAGAGGCTGCACTCCACAG −3′ (sense), 5′-GCTCACTCAGGGTCAGGTTG-3′ (antisense)], eNOS [5′-ATGGATGAGCCAACTCAA-3′ (sense), 5′-ATGGATGAGCCAACTCAA-3′ (antisense)] and glyceraldehyde 3-phosphate dehydrogenase (GAPDH) [5‘-GAGATGAGCT TCCTACAGCAC-3‘ (sense), 5′- TCATGAGGCCCTCCACGAT-3′ (antisense)]. Data analysis was carried out using Rotor Gene using the equation 2-Ct (cycle threshold): 2-[(Ct of target gene – Ct of GAPDH in treated dogs) – (Ct of target gene – Ct of GAPDH in sham dogs] [5].

### Statistical analysis

It was determined that six dogs in each arm would obtain a 90% study power, assuming 5% and 10% type I and II statistical errors, respectively, with expected 50% renal function preservation with sildenafil use [5]. The paired sample *t*-test was used to compare changes in means at different time intervals and the comparison of the changes between different groups was performed using repeated measure ANOVA. Comparison of median scores of renal degenerative scores, apoptotic markers and Nfr2 scores were performed by Mann–Whitney *U*-test. Inflammatory cytokines and eNOS mRNAs in renal tissue were expressed as means (SDs) and compared using independent sample *t-*tests. Statistical analysis was performed using the IBM Statistical Package for the Social Sciences (SPSS®), version 21 (SPSS Inc., IBM Corp., Armonk, NY, USA), with *P* < 0.05 considered statistically significant.

## Results

### Renal function changes

**Figure 2.**
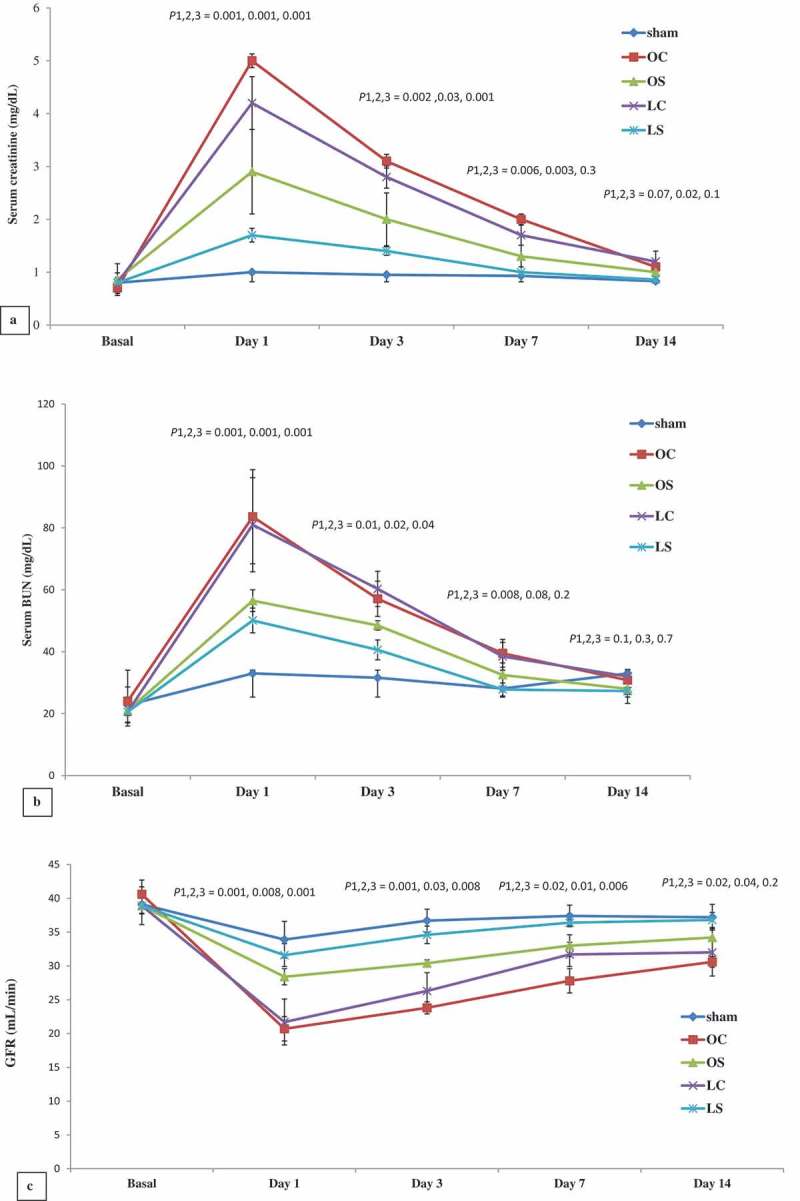
Mean (SD) serum creatinine (a), BUN (b), and GFR (c). *P*1 = OS group compared to OC group, *P*2 = LS group compared to LC group, and *P*3 = LS group compared to OS group.

Serum creatinine, BUN and GFR showed no statistical significant difference amongst all studied groups at baseline. The endpoint values of serum creatinine and BUN were significantly increased in all groups (*P* < 0.05) in comparison to baseline values. Similarly, GFR decreased significantly compared to its baseline value (*P* < 0.05). In comparison the sham group and control groups (OC and LC) showed statistically significantly higher levels of serum creatinine and BUN with lower GFR at the endpoints of the study. The sildenafil-treated groups (OS and LS) showed statistically significantly lower serum creatinine and BUN with higher GFR than their controls (OC and LC) (*P* < 0.05). On comparison of the sildenafil-treated groups, the LS group had statistically significantly lower serum creatinine and BUN and higher GFR at 1 and 3 days (Figure 2 (a–c)).

### Renal histopathology

**Figure 3.**
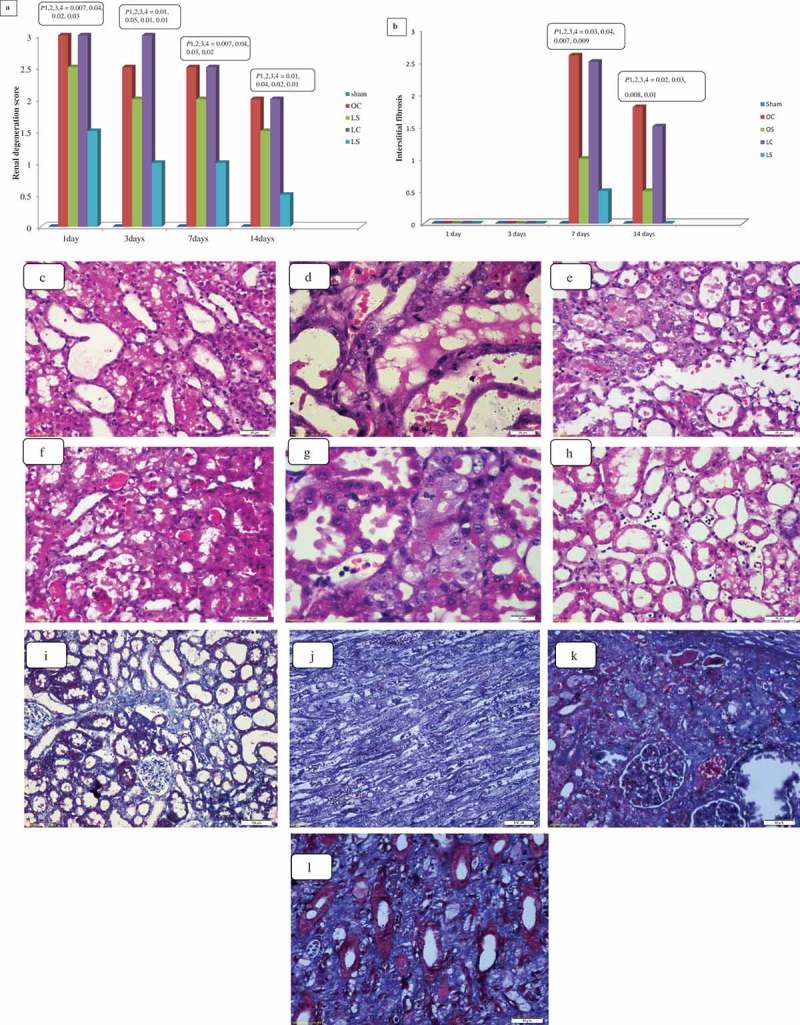
Scores of tubule-interstitial damage (a) and interstitial fibrosis (b) in kidney tissues of different groups at different time intervals; and illustrative figures of the outer medulla showing dilated irregular tubules with attenuated tubules in the LC group (c), cellular casts in the OC group (d), medulla necrotic tubules in the OS group (e), mild tubular injury with small casts at the LS group (f), the cortex of the kidney showing regenerating tubules with prominent nucleoli with mitotic figures in the LS group (g), and moderate neutrophil infiltrates in the OC group (h) at day 3 (H&E, ×400); and illustrative figures of the inner cortex showing minimal fibrosis in the OC group (i), fibrosis in the LC group (j), and superficial cortex showing fibrosis in the OS group (k) and outer medulla showing interstitial fibrosis in the LS group (l) at 14 days (Masson trichrome, ×400). *P*1 = control groups compared to sham group, *P*2 = OS group compared to OC group, *P*3 = LS group compared to LC group, and *P*4 = LS group compared to OS group.

The renal degeneration score decreased significantly at days 7 and 14 (*P* < 0.05) in comparison to day 1 in the control groups. Whilst, in the sildenafil-treated groups, it decreased significantly at 3, 7 and 14 days (*P* < 0.05) compared to day 1. The sham group showed statistically significant lower cortical and medullary damage scores than the other groups (*P* < 0.05). In comparison to the control groups, the sildenafil-treated groups showed statistically significantly lower renal damage scores (*P* < 0.05). On comparison of both sildenafil-treated groups, the LS group had a statistically significantly lower renal damage score (*P* < 0.05; Figure 3 (a, c–i)).

For interstitial fibrosis, there was a significant increase in the score at days 7 and 14 (*P* < 0.05). Compared to day 7, there was a statistically significant decrease in the score in all groups (*P* < 0.05). The sildenafil-treated groups showed statistically significantly lower cortical and medullary interstitial fibrosis than the control groups after 7 and 14 days (*P* < 0.05), with statistically significant differences in favour of the LS group (Figure 3 (b, j–l)).

### Renal apoptosis

**Figure 4.**
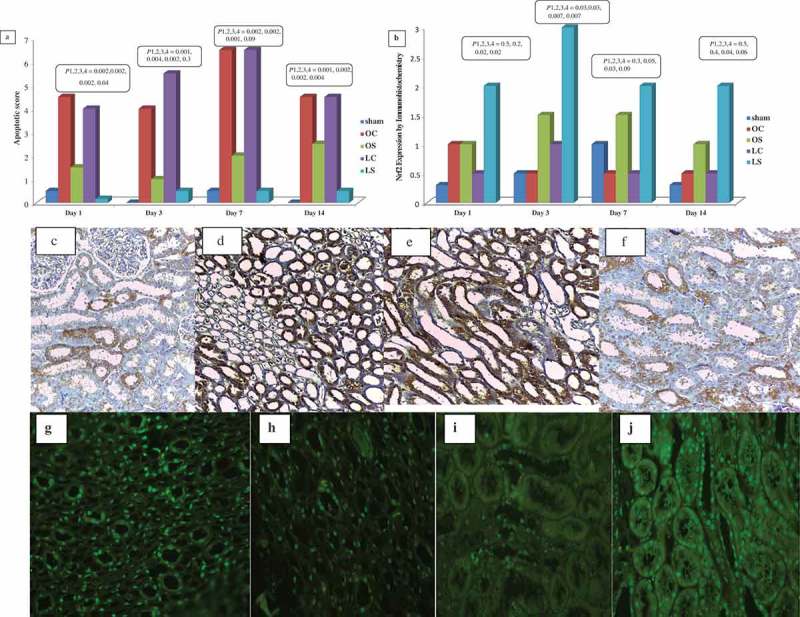
Shows the scores of caspases-3 expression of caspase-3 by immunohistochemistry (a) and Nrf2 by immunofluorescence (b) in kidney tissues and illustrative figures of mild expression of caspases-3 in the LS group (c), marked expression of caspases-3 in the LC group (d), marked expression of caspases- 3 in the OC group (e) and moderate expression of caspases-3 in the OS (f) (×400); and illustrative figures of moderate nuclear expression of Nrf2 in the LS group (g), mild-to-moderate nuclear expression of Nrf2 in the LC group (h), minimal-to-mild nuclear expression of Nrf2 in the OC group (i), and mild-to-moderate nuclear expression of Nrf2 in the OS group (j) (×400). *P*1 = control group compared to sham group, *P*2 = OS group compared to OC group, *P*3 = LS group compared to LC group, and *P*4 = LS group compared to OS group.

In the control groups, caspase-3 significantly increased at day 7 compared to day 1 (*P* < 0.05) and then it decreased insignificantly at day 14 (*P* > 0.05). In the sildenafil-treated groups, there was an insignificant rise in caspase-3 expression compared to day 1 (*P* > 0.05). In comparison to the sham group, all other groups showed statistically significantly higher expression of caspase-3. The sildenafil-treated groups showed statistically significantly lower expression of caspase-3 in comparison to their control groups, with lower expression in the LS group than OS group that reached statistical significance at 1 and 14 days after renal IR injury (*P* ≤ 0.04; Figure 4 (a, c–f)).

### Renal Nrf2 expression

Compared to day 1, there was an insignificant rise in Nrf2 expression in all groups at days 3, 7 and 14 except for the LS group, which had a significant rise at day 3 (*P* = 0.04). The sildenafil-treated groups showed statistically significantly higher expression of Nrf2 than their controls, whilst the expression in the LS group was statistically significantly higher than the OS group at days 1 and 3 after IR injury (*P* ≤ 0.02; Figure 4 (b, g–j)).

### The expression of mRNAs of inflammatory cytokines (TNF-α, IL-1β, ICAM-1) and eNOS

The expressions of mRNAs of inflammatory cytokines were statistically significant lower in the sildenafil-treated groups than the control groups at all time intervals (*P* < 0.05) and this was more in the LS group compared to the OS group at the different time intervals (*P* < 0.001). Conversely, the expression of eNOS mRNA was statistically significantly higher in the sildenafil-treated groups than the control groups. On comparison of both sildenafil-treated groups, the LS group had statistically significantly higher eNOS mRNA expression than the OS group at the different time intervals (*P* < 0.001; Table 1).

**Table 1.** Expression of pro-inflammatory cytokines and eNOS mRNA in the different groups.

Gene expression, mean (SD)#	Day 1	Day 3	Day 7	Day 14
TNF-α				
Sham	1.4 (0.28)	0.3 (0.3)	1 (0.3)	1.4 (0.4)
OC	14.7 (0.4)	13.6 (0.3)	12.6 (0.35)	11.3 (0.4)
OS	10.2 (0.4)	9.6 (0.29)	8.8 (0.3)	7.9 (0.3)*
LC	11.4 (0.6)	10.1 (0.3)	8.8 (0.5)*	9 (0.6)*
LS	2.8 (0.4)	2.3 (0.4)	1.6 (0.18)*	1.1 (0.1)*
*P* value (*P*1, *P*2, *P*3, *P*4)	<0.001, 0.003, <0.001, <0.001	<0.001, <0.001, <0.001, <0.001	<0.001, 0.001, <0.001, <0.001	<0.001, 0.002, <0.001, <0.001
ICAM-1				
Sham	0.58 (0.29)	0.7 (0.3)	0.8 (0.2)	0.8 (0.3)
OC	6.6 (0.23)	5.4 (0.27)	4.4 (0.35)*	3.6 (0.2)*
OS	5.3 (0.45)	4.4 (0.4)	3.7 (0.2)*	2.4 (0.3)*
LC	4.4 (0.3)	3.6 (0.3)	2.6 (0.3)*	1.4 (0.2)*
LS	3.5 (0.3)	2.4 (0.2)*	1.6 (0.24)*	0.8 (0.17)*
*P* value (*P*1, *P*2, *P*3, *P*4)	<0.001, <0.001, <0.001, <0.001	<0.001, <0.001, <0.001, <0.001	<0.001, 0.004, <0.001, <0.001	<0.001, <0.001, <0.001, <0.001
IL-1β				
Sham	0.6 (0.28)	0.5 (0.26)	0.6 (0.3)	0.4 (0.2)
OC	5.8 (0.37)	4.7 (0.33)	3.6 (0.35)*	2.6 (0.2)*
OS	4.8 (0.3)	3.6 (0.4)	2.6 (0.3)*	1.6 (0.2)*
LC	3.7 (0.4)	2.6 (0.2)	1.8 (0.2)*	1.3 (0.18)*
LS	2.7 (0.3)	1.8 (0.3)	1.3 (0.18)*	0.6 (0.2)*
*P* value (*P*1, *P*2, *P*3, *P*4)	<0.001, 0.001, 0.001, <0.001	<0.001, <0.001, <0.001, <0.001	<0.001, <0.001, 0.001, <0.001	<0.001, <0.001, <0.001, <0.001
eNOS				
Sham	9.6 (0.5)	9.9 (0.9)	8.8 (0.9)	9 (0.1)
OC	1.8 (0.47)	3.2 (0.33)†	4.1 (0.27)†	5.2 (0.4)†
OS	3 (0.5)	4.3 (0.33)	5.4 (0.4)†	6.7 (0.4)†
LC	4.45 (0.5)	5.9 (0.5)	7.1 (0.3)†	8.3 (0.4)†
LS	5.4 (0.6)	6.8 (0.4)	8.6 (0.3)†	10.2 (0.6)†
*P* value (*P*1, *P*2, *P*3, *P*4)	<0.001, 0.003, 0.01, <0.001	<0.001, <0.001, 0.005, <0.001	<0.001, <0.001, <0.001, <0.001	<0.001, <0.001, <0.001, <0.001

*P*1, control group compared to sham group; *P*2, OS group compared to OC group; *P*3, LS group compared to LC group; *P*4, LS group compared to OS group. *Mann–Whitney *U*-test, †independent sample *t*-test.

## Discussion

In a previous study, systemic sildenafil attenuated the sequelae of IR injury in rats through enhancement of the expression of Nrf2 mRNA and protein in renal tubular cells; up-regulation of anti-oxidant and anti-apoptotic genes expressions with a significant reduction in pro-inflammatory cytokine expression [5]. However, systemic sildenafil is associated with systemic hypotension, which potentiates cortical vasospasm and discourages optimal perfusion after release of ischaemia. This limits its use in clinical trials including humans exposed to renal ischaemia [16]. Hence, is this renoprotective effect maintained if we use sildenafil in local renal perfusion or in humans?

Therefore, we designed a new model of renal IR injury in large animal, as a small animal model is not suitable for determining renal recoverability in humans. Also, human tolerance to renal ischaemia is reported to be closely parallel to large animal models [17–19]. A 60-min ischaemia was studied to produce sufficient renal IR injury for our test purposes and it is the maximum limits of warm ischaemia before significant and irreversible loss of renal function and dog mortality occur [18]. This model allowed perfusion of the kidney with the drug and allowed its drainage outside the body. Sildenafil was added to the perfusion fluid in the LS group in a dose equal to half the oral dose (0.5 mg/kg vs 1 mg/kg in OS group). This difference in the doses between OS and LS groups was to ensure sildenafil bioavailable equality in both groups [20,21]. This model of perfusion mimics the bench-side perfusion of the donor kidney in live-donor transplantation. Also, it allows proper assessing of the impact of induced ischaemia and the effect of the studied drug, and avoids the effect of other factors including surgical manipulation of the renal vessels, vascular anastomosis and uretero-neocystostomy effects. Thus, the effects of LS were compared with the effects of its systemic administration. The study endpoints were chosen to assess the effect of sildenafil at a relatively longer time to include the proposed two waves of apoptosis (first wave; 1–3 days, second wave: 1 week later) and delayed interstitial fibrosis [22].

We investigated the role of sildenafil in renal protection by assessing renal function changes. Serum creatinine and BUN are rough methods to estimate renal function. So, we assessed changes in GFR for better evaluation of renal function and consolidated our present results by histopathological examination of renal tissue. Administration of sildenafil significantly improved renal function indices in comparison to the control groups. The new finding in the present study is that LS intra-arterial administration showed better renal function than its systemic administration throughout the whole study and reached statistical significance in the first 7 days after renal IR injury. This is similar to other reports that solitary kidney function can be recovered to basal levels after 1 week of the onset of ischaemia. Also, compensatory renal growth and effective renal plasma flow of the solitary kidney is restored completely after 1 week [17].

Sildenafil administration was associated with better renal tubular damage scores than in the control groups. This was previously reported in rat models [5,8,9,23–25]. Similar results were reported in dogs after 15 and 30 min of warm ischaemia [26]. The LS group had a better renal tubular damage score than the OS group throughout the study period. Conversely, Hosgood et al. [6] reported that reperfusion of porcine kidneys with sildenafil had no significant improvement in renal morphology after 2 and 18 h of cold ischaemia. Our present results are similar to the reports of reduced cardiac infarct size with i.v. sildenafil administration in a cardiac model of IR injury in the rabbit [27]. Interstitial fibrosis is a sequale of renal ischaemia and one of the major factors contributing to renal impairment. Macrophage-derived TGF-β and platelet-derived growth factor β promote fibroblast proliferation and production of extracellular matrix and subsequently interstitial fibrosis [28]. LS showed significant improvement in interstitial fibrosis by the end of the study.

The inflammatory response plays a major role in renal injury caused by ischaemia. During renal ischaemia, renal endothelial and parenchymal cells release pro-inflammatory cytokines (TNF-α and IL-1β), chemokines, and activate complementary systems. This regulates expression of adhesion molecules like ICAM-1. The net result is recruitment, activation and sequestration of inflammatory cells that promotes endothelial leucocyte interaction, which releases more cytokines and ROS, and induces more injury [22]. Sildenafil is reported to reduce the expression of inflammatory cytokines in renal, hepatic and neural tissues. Also, it decreases inflammatory cell recruitment, infiltration and endothelial–leucocytic interaction [5,29,30]. We found that this anti-inflammatory effect was clearly greater in the LS group in comparison to the OS group, through a reduction in the expression of TNF-α, IL-1β and ICAM-1 mRNAs.

Renal apoptosis is one of the major pathophysiological changes in renal IR injury. Caspase-3 is the final step in the apoptotic cycle in response to renal ischaemia and initiates the morphological cascades of apoptosis. Sildenafil has been reported to decrease the number of apoptotic cells in remnant kidneys after renal ablation with 60-min ischaemia in rats [24]. In the present study, sildenafil was associated with statistically significantly lower caspase-3 expression, with LS having a greater anti-apoptotic effect than OS. The anti-apoptotic action of sildenafil is due to release of NO and upregulation of the NO pathway that results in increased expression of Bcl-2 protein. Activation of Bcl-2 protein down regulates Bax protein and inhibits activation of the intrinsic mechanism of apoptosis. This in turn decreases activation of caspase-3 [31]. Also, sildenafil increases NO expression in renal tissue and improves renal haemodynamics. This is through increasing the expression of eNOS mRNA and decreasing inducible NOS mRNA expression in renal tissue [8]. In the present study, sildenafil was associated with higher expression of eNOS mRNA, which was more prominent in the LS than OS group. The present study and our previous work confirm the anti-oxidant effect of sildenafil by the higher expression of Nrf2 [5]. Intra-arterial sildenafil is associated with statistically significantly higher expression of Nrf2 protein than its systemic use.

To the best of our knowledge, this is the first study to assess the effect of LS administration in renal perfusion during ischaemia in a model similar to live-donor renal transplantation. Although the ischaemia type is different, the confirmed reno-protective effect of sildenafil in the perfusion solution is a basis for further research involving live-donor renal transplantation. Additionally the renoprotective affect during warm ischaemia, which is more hazardous than cold, gives a glimmer of hope for its use in cold ischaemia during a transplantation procedure. Assessment of the drug effect was analysed over a relatively long period (2 weeks). This allowed proper evaluation of the recoverability of the renal function and assessment of the short- and long-term effect of the drug on renal ischaemia. Also, it reproduces similar results that were reported on rates and thus it excludes the presence of inter-species difference in response to sildenafil administration.

However, the present study has some limitations, the unavoidable manipulation and handling of the renal artery and the impact of the surgical procedure cannot be excluded. The effect of LS was not compared to the standard drugs used in the perfusion fluid during live-donor renal transplantation. Also, the immunological aspects of renal transplantation were not incorporated in the present model. Changes in systemic blood pressure were monitored during the procedure and i.v. fluid was maintained to prevent the hypotensive effect of sildenafil. However, the data regarding blood pressure changes are not available.

## Conclusion

Sildenafil provides a renoprotective effect against IR injury in a canine model via anti-inflammatory, anti-oxidant, and anti-apoptotic effects. Moreover, LS seems to be more effective than systemic sildenafil, especially in the early postoperative period. These results may provide a base for further research in to its use in perfusion solutions during live and cadaveric renal transplantation.
